# Alcohol and HIV Effects on the Immune System

**DOI:** 10.35946/arcr.v37.2.12

**Published:** 2015

**Authors:** Gregory J. Bagby, Angela M. Amedee, Robert W. Siggins, Patricia E. Molina, Steve Nelson, Ronald S. Veazey

**Affiliations:** Gregory J. Bagby, Ph.D., is Kai and Earl Rozas Professor in the Departments of Physiology and Internal Medicine; Angela M. Amedee, Ph.D., is associate professor in the Department of Microbiology, Immunology, and Parasitology; Robert W. Siggins, Ph.D., is assistant professor in the Department of Physiology; Patricia E. Molina, M.D., Ph.D., is Richard Ashman Professor and head of the Department of Physiology and director; and Steve Nelson, M.D., is dean of the School of Medicine and John H. Seabury Professor in the Departments of Internal Medicine and Physiology; all at the Alcohol and Drug Abuse Center of Excellence in the School of Medicine at Louisiana State University Health Sciences Center, New Orleans, Louisiana. Ronald S. Veazey, D.V.M., Ph.D., is professor in the Department of Pathology and Laboratory Medicine at the Tulane University School of Medicine, New Orleans, Louisiana, and chair of the Division of Comparative Pathology at Tulane National Primate Research Center, Covington, Louisiana.

**Keywords:** Alcohol consumption, alcohol use disorder, immune system, immune function, HIV, HIV infection, acquired immunodeficiency syndrome (AIDS), immune response, mucosal immune system, gastrointestinal tract, genital tract, lungs, CD4^+^ T cells, antiretroviral therapy

## Abstract

HIV disease and alcohol independently influence the human immune system, so it stands to reason that, together, their influence may be additive. Here, we review the evidence that alcohol can exacerbate HIV’s influence on the immune system, thereby affecting disease progression and transmission. We focus particularly on alcohol’s effect on the mucosal immune system in the tissues of the gastrointestinal tract, the genital tract and the lungs, all of which play a role in transmission and progression of HIV disease.

Alcohol use disorder (AUD) and HIV infection both affect the immune system and frequently coexist in the same person, potentially multiplying the risk of infectious disease. Infectious disease, in turn, continues to be a major health concern and leading cause of morbidity and mortality worldwide, despite major advances in our understanding of the immune system, improvements in sanitation practices, and use of antibiotics, vaccines, and antiviral drugs.

Infection with HIV is particularly troublesome for the immune system because it infects and destroys immune system cells called T helper lymphocytes or CD4^+^ T cells. Untreated, the disease progresses over a few years to AIDS, leading to eventual death for most people. The disease is transmitted from infected to uninfected people through biological fluids containing the virus, most commonly through sexual contact but also through contaminated needles and other means. Since its discovery in the early 1980s, HIV infection has become a pandemic, causing an estimated 36 million deaths. The World Health Organization estimates that in 2012, of the 35 million people living with HIV/AIDS (PLWHA), 2.3 million were newly infected and 1.6 million died of AIDS-related causes despite increased availability of effective antiretroviral therapy (ART) (Joint United Nations Programme on HIV/AIDS 2013).

AUD in the form of alcohol abuse and alcohol addiction are the most common and costly form of substance abuse in the United States and represent a global health problem. For PLWHA, rates of heavy drinking are even higher than those in the general population (Galvan et al. 2002). One study found that 82% of HIV-infected patients consumed alcohol, and half were classified as hazardous drinkers (Lefevre et al. 1995). Because AUD and HIV infection frequently coexist, studies have tried to understand the influence of alcohol consumption on the transmission and progression of HIV disease. For one, heavy alcohol consumption increases the risk of HIV transmission through its propensity to increase the likelihood of risky sexual behavior, including unprotected sex with multiple partners (Rehm et al. 2012; Shuper et al. 2009; Stall et al. 1986). However, as detailed at length in this issue, AUD also may affect innate immune defenses and adaptive immune responses and thereby potentially increase the likelihood of HIV transmission over and above alcohol’s known behavioral associations with infection risk. Once infected, studies find that PLWHA with AUD perform poorly at multiple levels of the HIV treatment cascade, including adherence to ART, resulting in a higher likelihood of virologic nonsuppression (Azar et al. 2010; Chander et al. 2006; Palepu et al. 2003). Large observational studies show that hazardous alcohol consumption decreases overall survival in PLWHA in what seems to be a dose-dependent manner (Braithwaite et al. 2007). In 2010, this journal devoted an issue to the many consequences of alcohol consumption on HIV transmission with an emphasis on prevention, HIV disease pathogenesis, progression and treatment, and the impact of alcohol–HIV comorbidity on the lung, liver, heart, and brain (Bryant et al. 2010).

Here, we focus on the impact of alcohol on the host defense response to HIV infection. In particular, we review the evidence that alcohol exacerbates HIV’s influence on the immune system and affects disease progression and transmission. In particular, we discuss alcohol’s effect on the mucosal immune system in the tissues of the gastrointestinal tract, the genital tract, and the lungs, all of which play a role in transmission and progression of HIV disease.

## Does Alcohol Interact With HIV?

Alcohol’s effect on the immune system already is complex, but it is made even more complex in the context of HIV disease. Alcohol either can be immunosuppressive or immune activating for the cells of the innate and adaptive immune systems (Molina et al. 2010; Szabo et al. 2009). Intoxicating doses of alcohol generally are immunosuppressive, which can lead to an increase in the incidence and severity of infections, especially pneumonia (Szabo et al. 2009; Zhang et al. 2008), and could result in increased secondary infections in HIV-positive patients. In contrast, chronic alcohol consumption can be immune activating, causing chronic inflammation and oxidative stress resulting in conditions such as alcoholic liver disease, acute respiratory distress syndrome, and muscle wasting.

Studies that directly examine the interaction between alcohol and HIV disease related to the immune system have found mixed results (Bagby et al. 1998; Hahn et al. 2010). Early studies by Basgasra and colleagues (1993) administered alcohol to people uninfected with HIV then isolated peripheral blood mononuclear cells from their blood and infected the cells with HIV. The alcohol increased HIV replication. In similar experiments, Cook and colleagues (1997) also observed this response but only in lymphocytes from a subset of individuals. Other early studies suggested that AUD accelerated disease progression. For example, Fong and colleagues (1994) reported that a user who heavily abuses alcohol rapidly progressed to AIDS shortly after seroconversion. That said, evidence of alcohol’s influence on blood CD4^+^ T cell counts in HIV-positive patients is mixed. For example, Pol and colleagues (1996) observed improved CD4^+^ cell counts after HIV-positive alcoholic patients who were not receiving ART stopped drinking. However, a recent study did not see an association between alcohol consumption and a change in CD4^+^ cell counts over time in the absence or presence of ART (Conen et al. 2013). And in a cross-sectional study of 325 participants, daily alcohol use did not statistically correlate with lower CD4^+^ cell counts among patients taking or not taking ART (Wu et al. 2011). In contrast, Baum and colleagues (2010) found that frequent alcohol users treated with ART were more likely to show a decline in CD4^+^ cell counts independent of baseline CD4^+^ cell count or HIV load. Likewise, this study revealed an increased likelihood of declining CD4^+^ cell counts in patients not on ART. Samet and colleagues (2007) also observed lower CD4^+^ cell counts in HIV patients not on ART, but heavy alcohol consumption by subjects on ART was not associated with lower CD4^+^ cell counts. A French study (Carrieri et al. 2014) following HIV infected subjects with access to ART found that low alcohol consumption, defined as less than 10 grams per day, was associated with higher CD4^+^ cell counts when compared with abstainers. In addition, participants in this study who were categorized as moderate alcohol consumers had CD4^+^ cell counts similar to abstainers. These investigators suggested that low alcohol consumption might be a proxy to healthier behaviors encompassing things like diet and exercise.

Reports on the effect of alcohol on viral load in patients on antiretroviral therapy also vary among studies. One study (Wu et al. 2011) found higher viral loads in HIV patients on ART who consume alcohol, whereas another study (Samet et al. 2007) did not. Patients who consume alcohol may have lower adherence to ART, resulting in higher levels of viremia (Baum et al. 2010). These higher viral loads, in turn, make patients more infectious during unprotected sex with uninfected partners, which becomes more likely when patients drink (Kalichman et al. 2013).

Epidemiological studies conducted prior to the use of ART failed to identify an effect of alcohol abuse on HIV disease progression (Coates et al. 1990; Kaslow et al. 1989). However, these studies faced many methodological obstacles, including difficulties obtaining accurate measures of alcohol use patterns and problems controlling for confounding factors such as variations in the HIV strain, patient age, demographics, ethnicity, time since infection, comorbidities, medication side effects, and a host of environmental influences. Researchers have gotten around these methodological issues by creating an animal model of HIV disease using simian immunodeficiency virus (SIV) in rhesus macaque monkeys (see textbox, “An Animal Model for Conducting Controlled Studies”).

## Alcohol, HIV, and the Mucosal Immune System

Many of the SIV studies examining the connection between alcohol, HIV, and the immune system have focused on the mucosal immune system, consisting of cells that reside in the tissues lining various parts of the body, particularly the gastrointestinal tract, genital tissue, and the lung. These areas represent a critical if not primary site of HIV infection. Further, converging evidence suggests that AUD influences mucosal tissue in a way that potentially increases HIV disease transmission and adversely affects disease pathogenesis, resulting in accelerated disease progression.

## The Gastrointestinal Mucosal Immune System

### Pathogenesis of Alcohol and HIV Infection

Both alcohol and HIV infection have profound and often overlapping adverse effects on the integrity and immunology of the gastrointestinal tract. It is now known that the intestinal tract plays a major role in the pathogenesis of HIV infection throughout all stages of infection, and increasing evidence suggests alcohol exacerbates many of these effects. Because examining intestinal immune responses in humans is difficult, determining the biologic effects of alcohol and HIV infection requires carefully controlled studies in relevant animal models.

**An Animal Model for Conducting Controlled Studies**

To conduct more controlled studies of alcohol’s biological effects on HIV disease, researchers have turned to studies in rhesus macaque monkeys infected with simian immunodeficiency virus (SIV), a credible animal model of HIV disease (Bagby et al. 1998). Discovered in the 1980s, SIV is structurally, biologically, and genetically related to HIV. Like HIV, it enters host cells through CD4^+^ surface proteins and causes CD4^+^ T cell depletion, resulting in immunodeficiency that progresses to an AIDS-like state with opportunistic infections, muscle wasting, and neurological problems. This model provides many experimental advantages to studies in humans. It allows researchers to:

Control the timing of infection, the route of infection, the dose of SIV, and the strain of SIV;

- Control the dose and timing of alcohol delivery prior to and during infection;
- Control the experimental variables such as nutrition, drug delivery, and behavior;
- Perform longitudinal studies from the earliest events through end-stage disease as a result of shortened duration relative to the clinical course in people living with HIV/AIDS (PLWHA) (median survival is less than 1 year with pathogenic strains of SIV); and
- Perform studies in either the absence or presence of antiretroviral therapy (ART).

With regard to alcohol delivery, in our alcohol–SIV model, we provide rhesus macaques (Macaca mulatta) with a nutritionally balanced primate chow supplemented with fruit and ad libitum water. Starting 3 months prior to inoculation with SIV and continuing for the duration of the study, the macaques receive either ethanol or sucrose via a gastric catheter (Bagby et al. 2003). This alcohol delivery approach is chosen to ensure each animal receives similar alcohol doses and achieves similar circulating alcohol concentrations. We use two alcohol delivery protocols to simulate chronic binge alcohol (CBA) consumption: 5-hour infusion sessions 4 days per week, or half-hour infusion sessions 7 days per week. Individual doses are adjusted to achieve an intoxicating plasma alcohol concentration of 0.23 to 0.27 percent measured 2 hours after initiating alcohol delivery in the daily protocol and 5 hours after initiating alcohol delivery in the 4-day-per-week protocol. These protocols model heavy chronic binge alcohol consumption, which is the most common pattern of excessive alcohol use in the United States (Centers for Disease Control and Prevention 2012). With both protocols, animals received 13 to 14 g of alcohol per kilogram per week. Animals are inoculated with SIV either intravenously or intrarectally. As a rule, SIV infection leads to a peak viral load after about 2 weeks, which then decreases over the next few weeks to a steady-state termed viral “set point.” A high viral set point indicates that an animal will progress more rapidly to end-stage disease compared with animals with low viral set points (Staprans et al. 1999).

Overall, studies of this kind find that CBA increases virus production in tissue and plasma of SIV-infected animals (Bagby et al. 2006; Kumar et al. 2005; Nelson et al. 2013; Poonia et al. 2005). In two separate studies (Bagby et al. 2006; Nelson et al. 2013), CBA/SIV infected rhesus macaques had higher viral set points than sucrose controls, and progressed faster to end-stage disease with a median survival time of 374 versus 900 days, respectively (Bagby et al. 2006). As expected, blood CD4^+^ lymphocyte numbers decreased during SIV infection but did not differ between CBA and sucrose-treated animals. Another study, however, found a positive correlation between plasma viral load and blood CD4^+^ lymphocytes (mostly naïve T cells) in CBA but not sucrose-treated animals (Pahar et al. 2013).

These studies allow us to ask specific questions about the connection between alcohol, HIV disease, and the immune system and how those connections may influence the transmission and progression of HIV disease.

ReferencesCenters for Disease Control and Prevention (CDC)Vital signs: Binge drinking prevalence, frequency, and intensity among adults: United States, 2010MMWR: Morbidity and Mortality Weekly Report611419201222237031BagbyGJStoltzDAZhangPSIV infection, alcohol and host defenseAlcoholism: Clinical and Experimental Research22193S195S199810.1111/j.1530-0277.1998.tb03999.x9727633BagbyGJStoltzDAZhangPThe effect of chronic binge ethanol consumption on the primary stage of SIV infection in rhesus macaquesAlcoholism: Clinical and Experimental Research27495502200310.1097/01.ALC.0000057947.57330.BE12658116BagbyGJZhangPPurcellJEChronic binge ethanol consumption accelerates progression of simian immunodeficiency virus diseaseAlcoholism: Clinical and Experimental Research3017811790200610.1111/j.1530-0277.2006.00211.x17010145KumarRPerez-CasanovaAETiradoGIncreased viral replication in simian immunodeficiency virus/simian-HIV-infected macaques with self-administering model of chronic alcohol consumptionJournal of Acquired Immune Deficiency Syndromes3938639020051601015710.1097/01.qai.0000164517.01293.84NelsonSHappelKIZhangPEffect of bacterial pneumonia on lung simian immunodeficiency virus (SIV) replication in alcohol consuming SIV-infected rhesus macaquesAlcoholism: Clinical and Experimental Research37969977201310.1111/acer.12070PMC366170723414480PaharBAmedeeAMThomasJEffects of alcohol consumption on antigen-specific cellular and humoral immune responses to SIV in rhesus macaquesJournal of Acquired Immune Deficiency Syndromes6433234120132379941110.1097/QAI.0b013e31829f6dcaPMC3812314PooniaBNelsonSBagbyGJChronic alcohol consumption results in higher simian immunodeficiency virus replication in mucosally inoculated rhesus macaquesAIDS Research and Human Retroviruses2186386820051622541310.1089/aid.2005.21.863StapransSIDaileyPJRosenthalASimian immunodeficiency virus disease course is predicted by the extent of virus replication during primary infectionJournal of Virology734829483919991023394410.1128/jvi.73.6.4829-4839.1999PMC112526

Early studies in the SIV model demonstrated that, regardless of the route of infection, the intestinal tract is the primary target for SIV replication, amplification, and marked CD4^+^ T cell depletion in the earliest stages of infection (Veazey et al. 1998). These studies have been confirmed in HIV-infected humans (Brenchley et al. 2004; Mehandru et al. 2004), and the role of the gastrointestinal tract has since been a major focus of HIV research (Sandler et al. 2012). Indeed, research shows that, within the first few days of HIV infection, the virus rapidly infects and eliminates most of the CD4^+^ T cells in the intestinal tract (Sandler et al. 2012). Because the gastrointestinal tract contains most of the lymphocytes in the body, this represents a profound loss of total CD4^+^ T cells in the body (Douek 2007; Picker 2006). To best understand the interaction of alcohol and HIV on the intestinal immune system, we must first briefly explain the structure and compartmentalization of immune tissues in the gut. A more in-depth description of the gastrointestinal tract and a review of its immune system, also known as the gut-associated lymphoid tissue (GALT), is described by Hammer and colleagues, in this issue.

GALT comprises both specialized regions of lymphoid tissues, called Peyer’s patches, and more diffuse lymphoid tissues scattered throughout the layer of the intestinal wall called the lamina propria. The specialized regions provide immune surveillance for the intestines and tend to initiate the gastrointestinal immune response. There is an even larger pool of differentiated T cells, called CD4^+^CCR5^+^ T cells, and plasma cells diffusely scattered throughout the intestine’s lamina propria that serve as the “effector” arm of the intestinal immune system, actively battling antigens first encountered by the Peyer’s patches (Mowat et al. 1997).

HIV/SIV infection targets and eliminates these activated effector CD4^+^CCR5^+^ T cells, which are crucial for providing help for essentially all major innate and adaptive immune responses in the gut including cytotoxic functions, cytokine regulation, and controlling the production and secretion of immunoglobulins, particularly IgA (Xu et al. 2013). The viruses also selectively and rapidly infect and eliminate key subsets of CD4^+^ T cells that recent research shows are critical for the gastrointestinal immune response. Specifically, HIV and SIV target CD4^+^ T cells that produce interleukin (IL)-17 (Th17) or IL-22 (Th22) (Brenchley et al. 2008; Cecchinato et al. 2010), which seem to be crucial for regulating innate immune responses, especially the development and maintenance of the GALT (Ouyang et al. 2008; Paiardini 2010). These findings suggest that selective targeting and elimination of key subsets of CD4^+^ T cells in the intestine by HIV/SIV can have a profound influence on intestinal barrier function and adverse effects on both T- and B-cell responses in the gut (Klatt et al. 2010). Because alcohol also disrupts intestinal barriers and reduces Th17 cells (Asquith et al. 2014), it is likely that alcohol use in HIV patients results in even greater levels of intestinal barrier damage, leading to the leakage of intestinal microbes outside of the intestines—what is called “microbial translocation”— which can lead to inflammation and infections.

### Microbial Translocation in Alcohol and HIV

Both alcohol consumption and HIV/SIV infection seem to disrupt the intestinal lining, disrupt intestinal barrier function, and lead to microbial translocation. Indeed, both short- and long-term alcohol consumption can cause intestinal barrier permeability and movement of luminal bacterial antigens into systemic circulation through the blood, resulting in chronic inflammation and eventually liver damage (Elamin et al. 2013; Parlesak et al. 2000). Research also finds microbial translocation in the blood of HIV patients and SIV-infected macaques in the absence of alcohol as evidenced by the presence of lipopolysaccharide (LPS) endotoxins (Brenchley et al. 2006*b*; Klatt et al. 2010; Sandler et al. 2012). In fact, bacteria leaking through damaged intestinal barriers as a result of HIV-induced destruction of the key regulatory CD4^+^ T cells seems to be responsible for the systemic immune activation that is a hallmark of HIV infection (Brenchley et al. 2006*b*; Klatt et al. 2010; Sandler et al. 2012) and is a better predictor of HIV disease than plasma viral loads (Deeks et al. 2004). Indeed, levels of systemic activation appear to play a major role in the susceptibility to infection (Giorgi et al. 2002; Klatt et al. 2010, 2013) and disease progression (Deeks et al. 2004; Klatt et al. 2010).

Together, these findings suggest that the adverse effects of alcohol and HIV infection on microbial translocation may be additive. In addition, the inflammation and epithelial cell barrier damage that alcohol and its byproducts inflict on the intestinal mucosa (Elamin et al. 2013) may result in greater levels of mucosal and systemic immune activation, rendering patients more vulnerable to HIV transmission.

### Microbial Flora and Innate Mucosal Immunity in HIV and Alcohol Abuse

It is increasingly clear that the intestinal microflora play a role in the health of the gastrointestinal immune system. Research clearly shows that alcohol use alters the normal intestinal microflora, leading to intestinal damage and increased levels of LPS leaking into the blood where it circulates throughout the body (Elamin et al. 2013). Together, this suggests that alcohol use in HIV patients may influence the rate of intestinal T cell turnover and innate mucosal immune responses.

As with microbial translocation, the combination of HIV and alcohol may have an additive effect on the intestinal microbiome. HIV infection alone is associated with changes in the intestinal microbiome of infected patients, and those changes may be linked to HIV-induced alterations in the mucosal immune system (McHardy et al. 2013; Saxena et al. 2012). Although less studied, the combined effects of alcohol use and HIV infection are likely to induce marked changes in the intestinal microflora, and the innate immune responses in the gut, which should be a focus of future studies.

### Intestinal T Cell Turnover in HIV Infection

As suggested in the preceding sections, SIV and HIV infection lead to chronic immune activation, which creates a pro-inflammatory “cytokine storm.” This storm recruits and activates additional CD4^+^ T cells into mucosal tissues, providing the virus with a continuous supply of activated memory CD4^+^ T cells to infect (Mogensen et al. 2010; Wang et al. 2013). Continued activation, recruitment, and turnover of these viral target cells in damaged mucosal tissues may provide the “fuel” for continued viral replication and persistence in the gut. Indeed, it is increasingly clear that the intestinal tract is one of the major reservoirs for viral persistence of SIV in macaques (Ling et al. 2010) and of HIV in humans, even those on antiretroviral therapy (Avettand-Fenoel et al. 2011; Poles et al. 2006). One study found that alcohol increases the percentages of memory CD4^+^ T cells in the gut, suggesting that alcohol use may increase turnover of viral target cells in intestinal tissues (Poonia et al. 2006). Although the mechanisms behind this still are under investigation, it is possible that repetitive damage to the intestinal epithelium by alcohol may simply result in chronic inflammation, which recruits these T cells to the intestine through the above pathways. Thus, alcohol use in HIV infection may result in increased turnover of viral target cells in the intestine, which may partially explain why macaques infected with SIV have significantly higher levels of viremia in primary SIV infection (Bagby et al. 2006; Poonia et al. 2005).

Although more studies are needed to define the mechanisms involved, it is increasingly clear that HIV and alcohol use may have synergistic pathology, resulting in greater rates of disease progression in HIV patients, fueled by the loss of intestinal mucosal cells and chronic immune activation due to microbial translocation. Because many of these same processes may also occur in rectal and genital mucosal tissues, HIV and alcohol use may interact similarly to increase susceptibility to HIV infection and early replication following sexual transmission, a proposition we examine below.

### Alcohol and HIV Transmission via the Genital Tract

While HIV can be transmitted several ways, the primary method of transmission is through sexual contact. As such, it is critical to understand how AUD affects virus levels in genital fluids and the mucosal environment of the genital tract and rectum and how that may alter innate and adaptive immune responses and facilitate HIV transmission as well as susceptibility to infection. HIV levels present in the inoculating fluid—typically seminal or vaginal fluids—is a key factor in transmission of the virus from an infected individual to a noninfected host and typically is associated with HIV levels in the blood (Cohen et al. 2011). Because chronic alcohol abuse has been associated with increased plasma viral loads and more rapid disease progression (Baum et al. 2010; Rompalo et al. 1999), this population presents an increased risk for transmission. In female subjects, studies also have linked recent alcohol use with higher levels of virus in genital fluids when controlling for plasma HIV loads (Homans et al. 2012; Theall et al. 2008). These observations suggest that higher levels of HIV replication may occur in genital tissues of women that use alcohol, further increasing the potential for transmission. Similar studies in men have not been done.

Along with possibly promoting viral transmission, studies indicate that alcohol use by people who are uninfected may make them more susceptible to infection. In sexual transmission, HIV is acquired across the penile, vaginal, cervical, or rectal mucosa, and the integrity of the epithelial barrier and the innate defenses within these microenvironments provide the critical first lines of defense against HIV. The risk of HIV infection increases with increased inflammation within these local mucosal environments, likely as a result of the influx of HIV-susceptible cells and a potential breach in the epithelial barrier (Cohen 2004). Although no studies have examined in humans whether alcohol abuse can increase biological susceptibility to HIV infection, we recently found that chronic binge alcohol exposure that included intoxication at the time of exposure increased susceptibility to SIV infection (Amedee et al. 2014).

Studies have not directly evaluated the effects of chronic alcohol abuse on the genital mucosal environments. However, as detailed above, alcohol increases inflammation and the density of target CD4^+^ T cells in the upper gastrointestinal tract, making it feasible that it causes similar changes in barrier integrity and lymphoid cell levels in the lower areas of the gastrointestinal tract, which could facilitate HIV entry across the rectal mucosa. Chronic alcohol use may similarly affect the genital mucosa of the penis, vagina, and cervix through changes in target cell distribution and alterations of the innate defenses within the microenvironment. The early virus–host interactions following sexual exposure to HIV have been characterized most extensively in models of the female genital tract, and these studies describe rapid diffusion and penetration of the virus through the vaginal epithelium, followed by the formation of an initial focus of infected cells in submucosal tissues (Carias et al. 2013). These virus-infected cells move to draining lymph nodes, allowing the virus to spread systemically through the body (Li et al. 2009). Research has identified several innate factors in the vaginal compartment as critical first lines of defense in limiting HIV infection, including epithelial barrier integrity and antiviral proteins in vaginal fluids (Anderson et al. 2014; Cole 2006). In addition, the microbial flora of the vagina can directly influence these innate defenses by altering the function and integrity of the epithelial barrier and by influencing the inflammatory state of the compartment (Mirmonsef et al. 2012; Rose et al. 2012). Bacterial vaginosis, an infection characterized by an imbalance in the normal bacterial balance in the vagina and the absence of lactobacillus species in vaginal flora, has previously been associated with increased risk of HIV acquisition (Mirmonsef et al. 2012). In one study, women who reported alcohol use were less likely to have lactobacillus species present in their vaginal flora, leading to a flora consistent with that of bacterial vaginosis (Baeten et al. 2009).

Clearly, researchers need to conduct more directed studies to further define the specific alcohol-induced changes that alter innate defenses of the mucosal environments and that lead to an increased risk for HIV infection. As the numbers of HIV-infected persons continues to rise, it will become increasingly important to understand how chronic alcohol abuse affects genital virus expression and thus the risk of transmission. Additionally, the effects of alcohol on the genital microenvironment are important considerations in the development of pre-exposure prophylactic approaches. Alcohol-induced changes in microbial flora of the gut and vaginal compartments as well as changes to the epithelial barrier and innate defenses may alter the efficacy of antiviral approaches.

## Alcohol, HIV, and the Lung

The overlap between HIV and alcohol continues in another mucosal tissue—the lung. Both HIV and alcohol increase susceptibility to opportunistic infections, in particular, infection with bacteria that cause pneumonia. It is well established in humans and animal models that the immunosuppression caused by HIV infection frequently leads to pulmonary infections and that alcohol abuse impairs lung host defenses, resulting in a higher incidence and severity of pneumonia (Szabo et al. 2009; Young et al. 1989; Zhang et al. 2008), as detailed more specifically by Simet and Sisson, in this issue.

**Alcohol’s Influence on Wasting Disease**

Loss of muscle mass and what is called “wasting disease” is one hallmark of HIV in humans and SIV in rhesus macaques. Several studies suggest that chronic binge alcohol consumption (CBA) exacerbates this connection between muscle mass and SIV in part by triggering immune-related molecules. Although CBA- and sucrose-treated animals continued to gain weight and had comparable body weights during the first 300 days after SIV infection (Bagby et al. 2006), a closer evaluation of body composition revealed subtle but significant differences (Molina et al. 2006). For one, weight loss was a more common reason for euthanasia in alcohol-treated compared with sucrose-administered animals (Bagby et al. 2006; Molina et al. 2008), and muscle wasting was more pronounced in alcohol-consuming animals (Molina et al. 2008).

Chronic excessive alcohol consumption, even in the absence of SIV or HIV infection, alters the nutritional state, micronutrient availability, and tissue growth factor expression and activity (Molina et al. 2014). Indeed, approximately 50 percent of alcoholics show signs of alcoholic myopathy resulting from decreased muscle protein synthesis and accelerated breakdown of muscle proteins (Lang et al. 1999; Pacy et al. 1991; Preedy et al. 1994; Reilly et al. 1997; Teschner et al. 1988). Studies in SIV-infected rhesus macaques exposed to alcohol show that alcohol-mediated effects on muscle wasting are multifactorial, including decreased total caloric intake, altered nutrient selection, decreased nitrogen intake, and localized skeletal muscle inflammation and oxidative stress, which lead to a greater decrease in lean body mass and increase the incidence of AIDS (Molina et al. 2006, 2008).

Research in CBA/SIV macaques has begun to tease apart what is happening at the molecular and genetic level to breakdown the proteins that make up muscles and thereby decrease body mass. Simply put, within the skeletal muscle of CBA/SIV macaques there exists a molecular milieu that promotes the breakdown and inhibits the construction of new muscle.

The promotion of muscle breakdown seems to be regulated by the “ubiquitin-proteasome system (UPS),” which turns on any time muscles begin breaking down proteins because of conditions such as infections, burn injuries, fasting, and cancer (Fang et al. 1995; Hasselgren 1999; Llovera et al. 1994). The UPS is controlled by various molecules of the immune system, including glucocorticoids, catecholamines, and proinflammatory cytokines (Costelli et al. 1995; Price et al. 1994; Tiao et al. 1996). The genes encoding this system are stimulated in the skeletal muscle of emaciated AIDS patients (Llovera et al. 1998). In addition, studies in terminal stage SIV-infected rhesus macaques have shown a number of molecular markers related to muscle degradation, including significant increases in mRNA levels related to UPS, suppression of molecules such as insulin that promote muscle growth and increases in immune system molecules that promote inflammation (Molina et al. 2006, 2008). Similar imbalances in growth factors and pro-inflammatory molecules have been associated with wasting in AIDS patients (Franch et al. 2005; Nguyen et al. 1998). Studies in CBA/SIV macaques further confirmed the involvement of the UPS in muscle wasting, demonstrating that CBA increased molecules that hinder muscle growth and increased UPS activity in skeletal muscle in SIV-infected animals in the late stages of the disease. Moreover, those studies provided evidence of significant localized inflammatory milieu reflected in increased tumor necrosis factor (TNF)-α, interleukin (IL)-6, and IL-1β (LeCapitaine et al. 2011). Together, these findings suggest that inside the skeletal muscles of CBA/SIV macaques there exists a molecular milieu that favors the breakdown of muscle protein and suppresses its creation, while promoting a local proinflammatory state that compromises muscle health, leading to wasting.

ReferencesCostelliPGarcia-MartinezCLloveraMMuscle protein waste in tumor-bearing rats is effectively antagonized by a beta 2-adrenergic agonist (clenbuterol): Role of the ATP-ubiquitin-dependent proteolytic pathwayJournal of Clinical Investigation95236723721995773819910.1172/JCI117929PMC295859FangCHTiaoGJamesHBurn injury stimulates multiple proteolytic pathways in skeletal muscle, including the ubiquitin-energy-dependent pathwayJournal of the American College of Surgeons18016117019957850049FranchHAPriceSRMolecular signaling pathways regulating muscle proteolysis during atrophyCurrent Opinion in Clinical Nutrition and Metabolic Care827127520051580952910.1097/01.mco.0000165005.01331.45HasselgrenPORole of the ubiquitin-proteasome pathway in sepsis-induced muscle catabolismMolecular Biology Reports26717619991036365010.1023/a:1006916206260LangCHWuDFrostRAInhibition of muscle protein synthesis by alcohol is associated with modulation of eIF2B and eIF4EAmerican Journal of Physiology277E268E27619991044442210.1152/ajpendo.1999.277.2.E268LeCapitaineNJWangZQDufourJPDisrupted anabolic and catabolic processes may contribute to alcohol-accentuated SAIDS-associated wastingJournal of Infectious Diseases2041246125520112191789810.1093/infdis/jir508PMC3173505LloveraMGarcia-MartinezCAgellNUbiquitin gene expression is increased in skeletal muscle of tumour-bearing ratsFEBS Letters3383113181994830720010.1016/0014-5793(94)80290-4LloveraMGarcia-MartinezCAgellNUbiquitin and proteasome gene expression is increased in skeletal muscle of slim AIDS patientsInternational Journal of Molecular Medicine2697319989854146MolinaPEGardnerJDSouza-SmithFMAlcohol abuse: Critical pathophysiological processes and contribution to disease burdenPhysiology2920321520142478998510.1152/physiol.00055.2013PMC4046814MolinaPELangCHMcNurlanMChronic alcohol accentuates simian acquired immunodeficiency syndrome-associated wastingAlcoholism: Clinical and Experimental Research32138147200810.1111/j.1530-0277.2007.00549.xPMC317810618028526MolinaPEMcNurlanMRathmacherJChronic alcohol accentuates nutritional, metabolic, and immune alterations during asymptomatic simian immunodeficiency virus infectionAlcoholism: Clinical and Experimental Research3020652078200610.1111/j.1530-0277.2006.00252.x17117972NguyenBYClericiMVenzonDJPilot study of the immunologic effects of recombinant human growth hormone and recombinant insulin-like growth factor in HIV-infected patientsAIDS128959041998963114310.1097/00002030-199808000-00012PacyPJPreedyVRPetersTJThe effect of chronic alcohol ingestion on whole body and muscle protein synthesis: A stable isotope studyAlcohol and Alcoholism265055131991180413010.1093/oxfordjournals.alcalc.a045152PreedyVRSalisburyJRPetersTJAlcoholic muscle disease: Features and mechanismsJournal of Pathology1733093151994796539010.1002/path.1711730405PriceSREnglandBKBaileyJLAcidosis and glucocorticoids concomitantly increase ubiquitin and proteasome subunit mRNAs in rat muscleAmerican Journal of Physiology267C955C9601994794329110.1152/ajpcell.1994.267.4.C955ReillyMEMantleDRichardsonPJStudies on the time-course of ethanol’s acute effects on skeletal muscle protein synthesis: Comparison with acute changes in proteolytic activityAlcoholism: Clinical and Experimental Research2179279819979267527TeschnerMSchaeferRMWeissingerFChronic ethanol ingestion enhances catabolism and muscle protease activity in acutely uremic ratsNephron503383441988307041510.1159/000185199TiaoGFaganJRoegnerVEnergy-ubiquitin-dependent muscle proteolysis during sepsis in rats is regulated by glucocorticoidsJ ournal of Clinical Investigation97339348199610.1172/JCI118421PMC5070238567953

There are no reported clinical studies on the effect of AUD on the incidence and severity of opportunistic infections in PLWHA. And our longitudinal study of SIV infection to end-stage disease did not find more frequent secondary infections in alcohol-treated animals, nor were these infections, including pneumonias, a more likely underlying cause of end-stage disease in alcohol-treated animals (Bagby et al. 2006). This lack of a connection between alcohol and increased rate of lung infection may be attributed to the highly controlled biosafety practices used for these studies. Therefore, we set out to more fully address the influence of lung infection on host defense and SIV infection by measuring plasma and lung viral load in animals infected with pneumococcal bacteria load (Nelson et al. 2013). We chose pneumococcal infection because bacterial pneumonias are the most prevalent pulmonary complication in PLWHA (Wallace et al. 1993), and alcohol consumption is a well-known risk factor for bacteremic pneumococcal pneumonia in non–HIV- and HIV-infected populations (Nuorti et al. 2000).

We injected a sublethal dose of S. pneumoniae into a segment of the right lung of SIV-infected macaques. Twenty-four hours later, both alcohol-treated and sucrose control animals showed higher viral loads in bronchoalveolar lavage (BAL) fluid, which remained increased for at least 14 days in chronic alcohol-treated macaques. That said, we did not see differences between the alcohol-treated and sucrose control animals in plasma viral load, disease progression, or cytokine and neutrophil recruitment response. However, the early increase in local (lung) viral replication coincided with the host proinflammatory response that included nuclear factor (NF)-kB activation in recovered alveolar macrophages. The mechanism responsible for sustained increase in BAL fluid SIV RNA remains to be elucidated. Because macrophage NF-kB activation was limited to 1 day postinfection, it is possible that transient activation in alveolar macrophages along with the continued alcohol delivery is sufficient to sustain viral replication by these long-lived viral reservoir cells (Blankson et al. 2002). Alternatively, it is plausible that increased viral replication in alcohol-treated macaques occurs in other CD4^+^ cells in the lung or that NF-kB–independent mechanisms may be operant in pulmonary SIV replication during bacterial infection.

## Alcohol/HIV Interactions on the Hematopoietic System

Although most attention has been placed on HIV’s destruction of CD4^+^ T cells, AUD and HIV infection have been shown to independently cause defects in the normal formation of blood cells, a process known as hematopoiesis (Calenda et al. 1992; Yeung et al. 1988). Such changes potentially influence the replacement of cells that play a key role in innate and adaptive immunity. Prior to the development and use of effective ART, HIV patients commonly developed bone marrow pathologies, including dysplasia, impaired erythropoiesis, plasmacytosis, and lymphocytic infiltration into the marrow compartment (Calenda et al. 1992). Lymphocytic infiltration may represent cells recruited through inflammation, resulting in persistent local viral production in the bone marrow. In this regard, HIV has been shown to infect hematopoietic stem and progenitor cells (HSPCs) in a humanized mouse model (Nixon et al. 2013). Thus, bone marrow HSPC-containing proviral DNA may serve as a viral reservoir that, upon activation, may lead to increased local virus-mediated inflammation, further driving recruitment of lymphocytes that support more viral replication. Many of the antiretroviral drugs, including nucleoside reverse transcriptase inhibitors (NRTIs), non-NRTIs (NNRTIs), and protease inhibitors (PIs), largely correct these HIV-induced hematopoietic defects. However, side effects of these drugs are implicated in additional bone marrow pathologies (Baillou et al. 2003; Hernandez-Vallejo et al. 2013). In particular, ART seems to disrupt cells that serve a crucial role in guiding hematopoiesis. For example, PIs have been shown to promote aging and inhibit osteoblastic differentiation of mesenchymal stem cells, which may be a mechanism for reduced bone mineral density observed in PLWHA on ART (Hernandez-Vallejo et al. 2013; Yin et al. 2012). Binge alcohol administration also impairs osteoblastic differentiation, possibly contributing to dysregulated niche architecture. However, the combined effects of HIV, ART, and AUD on the bone marrow hematopoietic niche remain to be explored (Gong et al. 2004).

## Alcohol and Adaptive Immune Responses to HIV Infection

As described above, AUD has a significant impact on HIV disease through their direct and indirect pro- and anti-inflammatory effects. However, less is known about the effect of chronic alcohol use on the body’s specific, adaptive immune response to the virus and virally infected cells seen in both PLWHA and rhesus macaques infected with SIV. As discussed in more detail in the textbox, “Alcohol’s Influence on Wasting Disease,” chronic alcohol administration to rhesus macaques increases the plasma viral set point, which is associated with disease progression. These higher viral loads could be due to greater virus production coincident with immune activation or to impaired adaptive host defense.

Studies in SIV-infected macaques consistently show higher levels of viremia in animals receiving alcohol (Bagby 2003, 2006; Kumar 2005; Poonia 2006; Nelson 2013). In humans, alcoholics have been shown to have higher levels of blood T cell activation and higher proportions of memory CD4^+^ and CD8^+^ T cells (Szabo et al. 2009). Thus, higher viral loads in alcohol-receiving macaques is likely a reflection of higher turnover of viral target cells in mucosal tissues (Poonia et al. 2005). That said, specific adaptive immune responses to SIV do not seem to be impaired in animals receiving alcohol. In fact, we found that virus-specific CD8^+^ T cell responses in blood are even higher in alcohol-fed animals, which is likely a reflection of the higher viral loads in these animals (Pahar et al. 2013). Similarly, alcohol use seems to have little influence on viral-specific cellular immune responses in HIV/HCV coinfected patients (Graham et al. 2007).

Although a myriad of immunologic defects have been described in the blood of human drinkers (Szabo et al. 2009) and macaques (Siggins et al. 2009) receiving alcohol, the underlying mechanisms of these defects have yet to be elucidated. In addition, the blood is merely a “window” reflecting only a small part of the immune system as a whole, and the effects of mucosal barrier damage and inflammation may not be adequately reflected in monitoring peripheral blood alone.

## Conclusion

Both HIV and alcohol use clearly influence immune function, so it seems logical that, in tandem, they might have an additive influence on disease progression. In fact, research has begun to suggest that alcohol use by people infected with HIV can exacerbate an immune system that already is badly strained. The clearest connection between alcohol, HIV, and immune dysfunction is in the gastrointestinal tract where the damage alcohol does to the mucosal lining of the intestines leads to inflammation, barrier damage, and bacterial leakage, which in turn seems to strengthen HIV’s grip. Indeed, alcohol’s influence on the body’s many mucosal tissues seems to be synergistic with those of HIV, including the tissues of the intestines, the genital tract, and the lung, where chronic inflammation results in increased local and systemic viral replication, resulting in environments in mucosal tissues that both worsen disease progression, and increase the risks of viral transmission. Much more research remains to be done to clarify the interaction between and among alcohol, HIV, and the immune system and to elucidate the mechanisms involved in this complex interaction.
